# Editorial

**Published:** 1902-01-15

**Authors:** 


### EDITORIAL.

An amendment to the dental law of Ohio is to be urged at the present session of the legislature. The amendments sought are in importance; first, that none but graduates of a legally chartered dental college, recognized as reputable by the State board, shall be registered; and these only after the payment of a fee of twenty dollars, and passing a satisfactory examination before the board at its regular sessions in June and November. Except that graduates of Ohio schools shall be registered on payment of the fee of twenty dollars, without examination up to and including the November session of the board, 1904.

The second amendment is intended to more clearly define dental practice. It includes the manager, proprietor, or conductor of a dental office who performs dental operations on the teeth and jaws for a fee or its equivalent, and exempts dental students at work in college clinics, or when working for registered dentists in the interval of college sessions, providing they are attending consecutive sessions of a dental college. It also exempts physicians except when practicing dentistry as a specialty.

The third amendment is to provide reasonable compensation for the examiners and to authorize them to employ additional counsel in the prosecution of illegal practitioners.

The fourth amendment is to set the amount of fines to be imposed. For a first offense the fine may be fifty dollars and up to one hundred, or imprisonment for from ten to thirty days. For a second conviction the maximum penalties shall be inflicted.

If this bill can be passed it will certainly be a great improvement on the present law. It may seem somewhat oppressive in compelling all graduates to take examinations, but this should not be a great hardship to a man fresh from his studies, many States now require it, and more probably will. It may also seem rather discourteous to college faculties to have their work reviewed by an examining board, but we have no doubt it will stimulate all college faculties to bring their students up to a sufficiently high standard of attainment, that they can readily comply with this formality. It will be more severely felt by practitioners who have been long out of college, but such men will have large advantage in the technical departments, and so it would seem a fair measure to all.

There is no question but such laws are for the good of the commonwealth, as they will in time weed out the incompetent by supplanting them with a superior quality of practitioners, and the general public welfare in which behalf all such laws are enacted will be conserved. It is a very worthy bill and ought to be helped on by every proper means. Post your representative on its merits.

